# Alcohol Use in Adolescence and Risk of Disability Pension: A 39 Year Follow-up of a Population-Based Conscription Survey

**DOI:** 10.1371/journal.pone.0042083

**Published:** 2012-08-01

**Authors:** Anna Sidorchuk, Tomas Hemmingsson, Anders Romelsjö, Peter Allebeck

**Affiliations:** 1 Division of Social Medicine, Department of Public Health Sciences, Karolinska Institutet, Stockholm, Sweden; 2 Department of Epidemiology, Parasitology and Desinfectology, North-Western State Medical University named after I.I. Mechnikov, Saint-Petersburg, Russian Federation; 3 Division of Occupational and Environmental Medicine, Department of Public Health Sciences, Karolinska Institutet, Stockholm, Sweden; Research and Development Corporation, United States of America

## Abstract

**Background:**

The role of alcohol consumption for disability pension (DP) is controversial and systematic reviews have not established causality. We aimed to assess the role of adolescent alcohol use for future DP. We wanted to find out whether an increased risk mainly would affect DP occurring early or late in life as well as whether the level of alcohol consumption and patterns of drinking contribute differently in DP receiving.

**Methodology/Principal Findings:**

The study is a 39-year follow-up of 49 321 Swedish men born in 1949–1951 and conscripted for compulsory military service in 1969–1970. As study exposures (i) “risk use” of alcohol composed of measures related to pattern of drinking, and (ii) the level of consumption based on self-reported volume and frequency of drinking had been used. Information on DP was obtained from social insurance databases through 2008. “Risk use” of alcohol was associated with both “early DP” and “late DP”, i.e. granted below and above the approximate age of 40 years, with crude hazard ratio (HR) of 2.89 (95% confidence intervals (CI) 2.47–3.38) and HR of 1.87 (95%CI: 1.74–2.02), respectively. After adjustment for covariates, HR was reduced to 1.32 (95%CI: 1.09–1.59) and 1.14 (95%CI: 1.05–1.25), respectively. Similar patterns were seen for moderate (101–250 g 100% alcohol/week) and high (>250 g) consumption, though the risk disappeared when fully adjusted.

**Conclusions/Significance:**

Alcohol use in adolescence, particularly measured as “risk use”, is associated with increased risk of future DP. The association is stronger for “early DP”, but remains significant even for DP granted in older ages. Therefore, pattern of drinking in adolescent should be considered an important marker for future reduced work capacity.

## Introduction

The role of alcohol for disability pension (DP) still remains controversial. Timing of exposure and measure of consumption differ substantially between studies contributing to a high variability in study results over time and place. A 20-year follow-up of Swedish male military conscripts, found an increased risk of DP granted at the age below 40 among those reporting high alcohol consumption as well as problem drinking behavior during adolescence and young adulthood [1], [2]. On the other hand, a history of alcohol intoxication in adolescence was not related to future work incapacity in another Swedish study on female DP, while for those diagnosed with alcohol use/dependence during adulthood the association with DP was significant [3]. Exposure to alcohol in adulthood was also found to increase the risk of DP for males and females in the Stockholm Health of the Population [4] and in the Norwegian HUNT study [5]. The latter study, however, found only current problem drinking, but not high level of alcohol consumption, to be a strong predictor of work incapacity [5]. In all these studies alcohol measures were based exclusively on self-reported information that could have potentially biased the results. However, a Swedish study of middle-aged men, in which current alcohol overconsumption was measured both by questionnaires and a value of serum gamma glutamyl transferase, found an increased risk of DP for the exposed groups regardless of the manner of measurement [6]. Adjustment for health and life-style conditions, including socioeconomic position (SEP), often attenuate the risk of DP among drinkers [1], [2], [4], [6], but a possible causal relation can still exist. Another area of research is DP due to musculoskeletal disorders, in which some authors have assessed level of alcohol intake, but no effect of alcohol on DP was found [7]–[10].

Two systematic reviews [11]–[15] concluded that there is insufficient scientific evidence to establish a causal relationship partly due to a high variability in both exposure and outcome measures, though the EU report from 2011 [16] substantiated a positive association.

One of the reasons for the varying results is the complex interplay between a wide range of alcohol-related problems and DP as well as variations in criteria for granting DP [1]–[4], [6]. Another reason may be that different indicators of alcohol use reflect different aspects of the exposure [5], [17] and, therefore, contribute to variability in causal inference. Methodological issues may also contribute to inconsistency as it is rather difficult to differentiate between mediators and confounders, particularly if related to social, behavioral or psychological factors [5], [18]. The exact mechanisms of association between alcohol use and DP has not been yet clarified, though early life alcohol use may influence a certain “life career”, e.g. deviant behavior, low social adjustment, substance use, criminality, etc., and, therefore, prevent from getting or maintaining job [1], [2], [19].

What makes the issue even more intriguing is the fact that several studies reported abstainers to be at a higher risk of DP compared to light or moderate drinkers [4]–[6]. There has been several attempts to shed the light on associations between abstention and adverse health and behavioral outcomes [20]–[23], though results remain controversial and highly debated.

Sweden is among the countries with the highest prevalence of DP and the largest public spending on DP benefits [24]. During the last two decades a 3-fold increase has been reported in DP incidence among people aged below 30 [25]. In the mid-2000 s the new, more tighten rules for granting disability had been introduced that resulted in decline in total number of DP in Sweden after 2003–2004 [25]. In 2009, psychiatric diagnoses alone have accounted for more than 40% of newly granted DP, while in earlier time musculoskeletal conditions was the largest diagnostic group [25]. The Swedish legislation allowed alcohol dependence to be included in the list of the diagnoses for DP in 1977, but the proportion of DP with alcoholism as a main diagnosis has always been rather low, probably due to underreporting [26]. Due to a particularly high rate of psychiatric diagnoses for DP as well as to the fact that hazardous use of alcohol has been proven to result in a wide range of medical conditions limiting the working capacity, the concern over a possible role of alcohol in DP is increasing.

Earlier analyses of a cohort of Swedish men conscripted to military service in 1969–1970 showed an increased risk of DP up to the age of around 40 among those exposed to high alcohol consumption in late adolescence/early adulthood [1], [2]. Now spanning over almost 40 years life course we wish to contribute to further understanding how early life alcohol use affects future DP. Thus, we aimed to analyze the association between alcohol use in late adolescence and DP up 2008, i.e. to the age of around 59. We wanted to find out whether an increased risk mainly would affect DP occurring early or late in life as well as whether the role of alcohol use for future DP varies depending on which measure of alcohol use is applied: volume or pattern of drinking.

## Methods

### Ethics Statement

In several early applications to the Karolinska Institutet Ethical Review Board, we specifically pointed out that due to the character of the data base, it was impossible to trace persons and ask for written or verbal informed consent. Thus, the Institutional Review Board has waived the normal requirement for informed consent, since we only work on record linkage data that have been anonymized. Waiver was obtained in permissions Dnr 121/84 and Dnr 188/91, of which the permissions enumerated below are subsequent follow-ups.

### Study Population

Our study is a follow-up of the cohort of 50 465 Swedish men mandatory conscripted for military service in July 1, 1969- June 30, 1970. At the time only around 2–3% of men were exempted on account of severe mental or physical conditions. The present study includes 49 321 men (97.7% of original cohort) born in 1949–1951, i.e. aged 18–21 at conscription.

Detailed description of the cohort has been presented elsewhere [1], [22], [27]. In brief, at conscription all men answered two questionnaires. One questionnaire addressed family and social background, school performance, self-assessed health, psychological and behavioral factors and the other focused on use of alcohol, tobacco, various illicit drugs and sniffing of solvents. The total non-response rate was less than 1.2%. All men went through a medical examination with the variety of physical tests and were interviewed by a psychologist, who assessed conscript’s psychological status and sociability. Those who had symptoms or reported any mental disorders were examined by a psychiatrist and the diagnoses were recorded, according to the International Classification of Disease revision 8.

### Study Exposures

The *level of alcohol consumption* was based on self-reported information on average quantity and frequency of drinking of medium or strong beer, wine and strong spirits ever before conscription. The consumption in grams of 100% alcohol per week was calculated based on standard estimates of drink size (10–12 grams 100% ethanol) [28]. The conscripts were classified as: abstainers if they reported never drinking any of the beverages (0 g 100% alcohol/week), light (1–100 g), moderate (101–250 g) and high consumers (more than 250 g). The categories followed those previously used in studies of the same cohort [1], [22].

As the second exposure we used a composite variable *“risk use” of alcohol* as an indicator for hazardous drinking pattern. Subjects were classified as having “risk use” if at least one of the following conditions ever before conscription was fulfilled: having a history of being apprehended for drunkenness by the police, having taken an “eye-opener” (using a drink the first thing in the morning [29]) to overcome hangover, having been drunk often/quite often, reporting alcohol consumption of more than 250 g 100% alcohol/week [27], [30], [31].

### Study Outcome

Our study outcome was DP granted after the conscription and up to 2008. According to the National Insurance Act, DP may be granted to a person aged 16–65 whose working capacity is estimated to be permanently reduced as a result of physical or mental impairment, irrespective of whether the person is, or ever has been, employed [32]. The information on DP was obtained from the National Social Insurance Board for the period of 1971–1989 and from a Longitudinal Register of Education and Labor Market Statistics for 1990–2008 [32].

We categorized the outcome as “early DP” if received at the approximate age below 40, i.e. in 1971–1990, and “late DP”, i.e. after age of 40 in 1991–2008. The categories were similar to previous studies based on the same cohort [1], [2], [31] and reflected the changes in the rules for receiving DP introduced in the beginning of 1990 s [32].

The distribution of the exposure measures is presented in Table 1.

**Table 1:** Description of total cohort of 49 321 Swedish male conscripts born in 1949–1951 with respect to self-reported levels of alcohol consumption and different “risk use” of alcohol behaviors established at conscription in 1969–1970.

Alcohol habits	No of conscripts (%)
**Consumption of alcohol reported at conscription (g 100% alcohol/week)**	**49 321 (100)**
- Light consumers (1–100 g)	33 526 (68.0)
- Moderate consumers (101–250 g)	9 547 (19.4)
- Abstainers (0)	2 781 (5.6)
- High consumers (>250 g)	1 724 (3.5)
- No answer given^a^	1 743 (3.5)
**“Risk use” of alcohol composite variable**	**49 321 (100)**
- No “risk use” of alcohol	42 263 (85.7)
- “Risk use” of alcohol^b^	6 422 (13.0)
- Not established^c^	636 (1.3)

a Conscripts provided no answers to the questions on frequency and consumption level of beer, wine and spirit, therefore, the weekly level of consumption could not be calculated.

b Subjects were classified as having “risk use” if at least one of the following condition was fulfilled: having a history of being apprehended for drunkenness, needing an eye-opener to overcome hangover, having been drunk often/quite often, reporting alcohol consumption measured as more than 250 g 100% alcohol/week.

c Conscripts provided no answers to any of the questions to compose the variable “risk use” of alcohol of.

### Study Covariates

Based on earlier studies of this cohort [1], [2], [22], [27], [31], [33] and other studies on DP [3]–[5] we selected the following variables from the questionnaires: (1) *family background exposures* (father’s drinking habits (often, sometimes or rarely/never (reference group)), having divorced parents (yes *vs.* no)); (2) *school-related exposures* (truancy (once in semester or often *vs.* rarely), having ever taken remedial classes (yes *vs.* no)); (3) *social and behavioral exposures* (having been in contacts with police and childcare authorities (once or more *vs.* never), experience of ever running away from home (once or more *vs.* never), ever being unemployed more than 3 months after school (yes *vs.* no)); (4) *physical and mental health at conscription* (self-assessed health (very good/good, neither bad nor good or bad/very bad (reference group)), having ever taken medication for nervous problems (one or more *vs.* never), psychiatric diagnosis at conscription (yes *vs.* no)); (5) *psychological status and sociability at conscription* (emotional control (poor/very poor *vs.* very good/good/average) and social maturity (low/very low *vs.* very high/high/fair) [34], [35], cognitive ability (IQ) levels ranked on a Stannine scale (low (levels 1–3), moderate (levels 4–6) or high (levels 7–9) (reference group)) [36]); (6) *other substance use reported at conscription* (tobacco smoking (> than 10 cig/day, 1–10 cig/day or none (reference group)), sniffing of solvents (once or more *vs.* never), use of illicit drugs (ever *vs.* never)).

Information on family SEP was based on data on conscript’s father’s occupation in Census 1960: manual (unskilled, skilled), low non-manual, combined intermediate and high non-manual, and others (farmers, self-employed, unclassified).

### Follow-up and Missing Values

Register data combined for this study were provided by Statistics Sweden. Record linkages were made using the unique individual number for each conscript, which was substituted by Statistics Sweden for the original Swedish personal identification number, in order to ensure confidentiality of personal data. We performed linkages between the abovementioned conscripts’ cohort and Census 1960, the Total Population Register (migration status), the National Cause-of-Death Register (date of death), the National Social Insurance Board data sets and a Longitudinal Register of Education and Labor Market Statistics (DP status and date of DP granting).

Person-time (in all 1 779 132 person-years) was counted from October 1, 1969 for two subjects who died in 1969 and from January 1, 1970 for the rest of the conscripts until the date of receiving DP, date of death, date of emigration or until end of follow-up on the July 1, 2008.

Information from social insurance records was missing for 1.8% of conscripts. These men were censored at their last appearance in the population records. Only 3.5% of the conscripts refused to answer questions on frequency and levels of alcohol consumption. For 1.3% of the conscripts the variable “risk use” of alcohol could not be used.

### Statistical Analysis

The hazard ratio (HR) and 95% confidence intervals (CI) for DP in relation to the level of consumption and the “risk use” of alcohol was calculated by using Cox’ proportional hazards model. The proportional hazard assumption was checked by log survival plots. Analyses were conducted for total DP as well as for “early DP” and “late DP”.

Variables were included in multivariate analysis if found significant in univariate model and fulfilling the criteria for being confounders [37]. Some of the covariates, particularly related to social and psychological characteristics, mental health and sociability may mediate the pathway between alcohol use and DP [5], and result in overadjustment if controlled for. The explanatory variables had been measured only once, i.e. at conscription, and, therefore, time order was difficult to establish. Based on the nature of events as well as prior knowledge from the literature, we assumed that family background and school-related variables likely preceded the exposure and, therefore, might act as confounders. Variables reflecting social and behavioral characteristics, physical and mental health and other substance use might both precede and follow the exposure, i.e. might act as confounders or intermediates in the causal pathway. For example, contact with police and childcare authorities might take place due to the child being at risk in his environment (e.g. parental alcohol abuse) or to be a result of the child’s own behavior (e.g. child’s alcohol abuse). Variables related to psychological status and sociability might also both contribute to adolescent alcohol use, if self-medication hypothesis is applied [38]–[40], and result from it [19], [41], [42], therefore, the temporal order is not clear. Similarly to that, the onset of other substance use often co-occurs with early life alcohol drinking. Bi-directional relationship was reported for alcohol and tobacco smoking. Thus, alcohol was shown to predict smoking [43]–[45] and vice versa [46], though drinking was found to be a stronger predictor for smoking than the converse [47]. Alcohol and tobacco were seen to progress to subsequent use of illegal drugs [48]–[50].

Our uncertainties regarding the role of the abovementioned variables as potential confounders or mediators have been considered by applying different multivariate models as suggested by Rothman [37]. Thus, our **Model 1,** the most conservative, includes all study covariates. **Model 2,** a semi-conservative, includes family background and school-related variables as well as variables on psychological status and sociability. **Model 3,** the least conservative, includes only covariates preceding the exposure (family background and school-related variables). To ease the reporting, we grouped the variables according to the order they occurred during the life course in groups written in *Italic* in “Study covariates”.

We tested possible effect modification by baseline covariates using logrank test for stratification. The interaction HR was assessed separately for both exposure variables.

Information on the number of subjects included in the analysis at various stages is presented in Table 2. Only conscripts with complete information on all covariates were included. Thus, multilevel analyses included 38 671 (78.4%) persons to calculate HR for DP among different *alcohol consumption levels* with light consumers as a reference group, and 38 899 (78.9%) conscripts to calculate HR for DP among people with different history of *“risk use” of alcohol*.

**Table 2:** General description of the study cohort for 39-year follow-up (1969–2008).

Cohort description	N of persons (%)
Conscripts cohort 1969–1970 (born in 1949–1951)	49 321 (100)
Number of persons granted DP in 1971–2008, according to RFV^a^, LOUISE^b^/LISA^c^ databases	6 342 (12.9)
Among them:	
- “Early DP” granted in 1971–1990	1 038 (2.1)
- “Late DP” granted in 1991–2008	5 304 (10.7)
Number of persons not granted DP in 1971–2008 according to RFV^a^, LOUISE^b^/LISA^c^ databases	38 767 (78.6)
Died during follow-up	2 469 (5.0)
Emigrated	844 (1.7)
Lost to follow-up (no information on DP status)	899 (1.8)
Number of conscripts not answering questions on alcohol consumption level (exposure I) in the survey	1 743 (3.5)
Number of conscripts not answering any of the four key questions to form a composite variable “risk use” of alcohol(exposure II)	636 (1.3)
Number of conscripts included in the final analysis of alcohol consumption level at conscription and DP, i.e. withinformation available for all covariates	38 671 (78.4%)
Number of conscripts included in the final analysis of “risk use” of alcohol and DP, i.e. with information availablefor all covariates	38 899 (78.9%)

Abbreviations: DP, Disability Pension.

a The National Swedish Social Insurance Board database.

b The Longitudinal Register of Education and Labor Market Statistics.

c The Longitudinal Database Integration for Medical Insurance and Labor Studies.

To address the issue of potential heterogeneity among those reporting abstention from alcohol we ran a series of additional analyses. First, we divided the group of abstainers in two sub-groups by including those who abstained from alcohol and all other substance use, i.e. tobacco smoking, sniffing of solvents and illicit drug use, in a sub-category of “pure abstainers” leaving those who abstained exclusively from alcohol, but not from the other substances in a sub-category of “other abstainers”. Second, we performed a frequency analysis to study the relation between different categories of abstention and other medical, social and behavior characteristics. Third, we repeated the main analysis by assessing HR and 95% CI for DP in relation to different levels of consumption, including both sub-categories of abstainers. Finally, we ran an additional analysis for association of DP with “risk use” of alcohol in a sub-cohort excluding abstainers.

We checked the robustness of our results in sensitivity analyses by repeating the main analyses including all 49 321 conscripts. All reported *P*-values are two-sided. *P*<0.05 was considered statistically significant. Statistical analyses were performed using STATA version 11 (StataCorp, College Station, TX).

## Results

**Figure 1:**
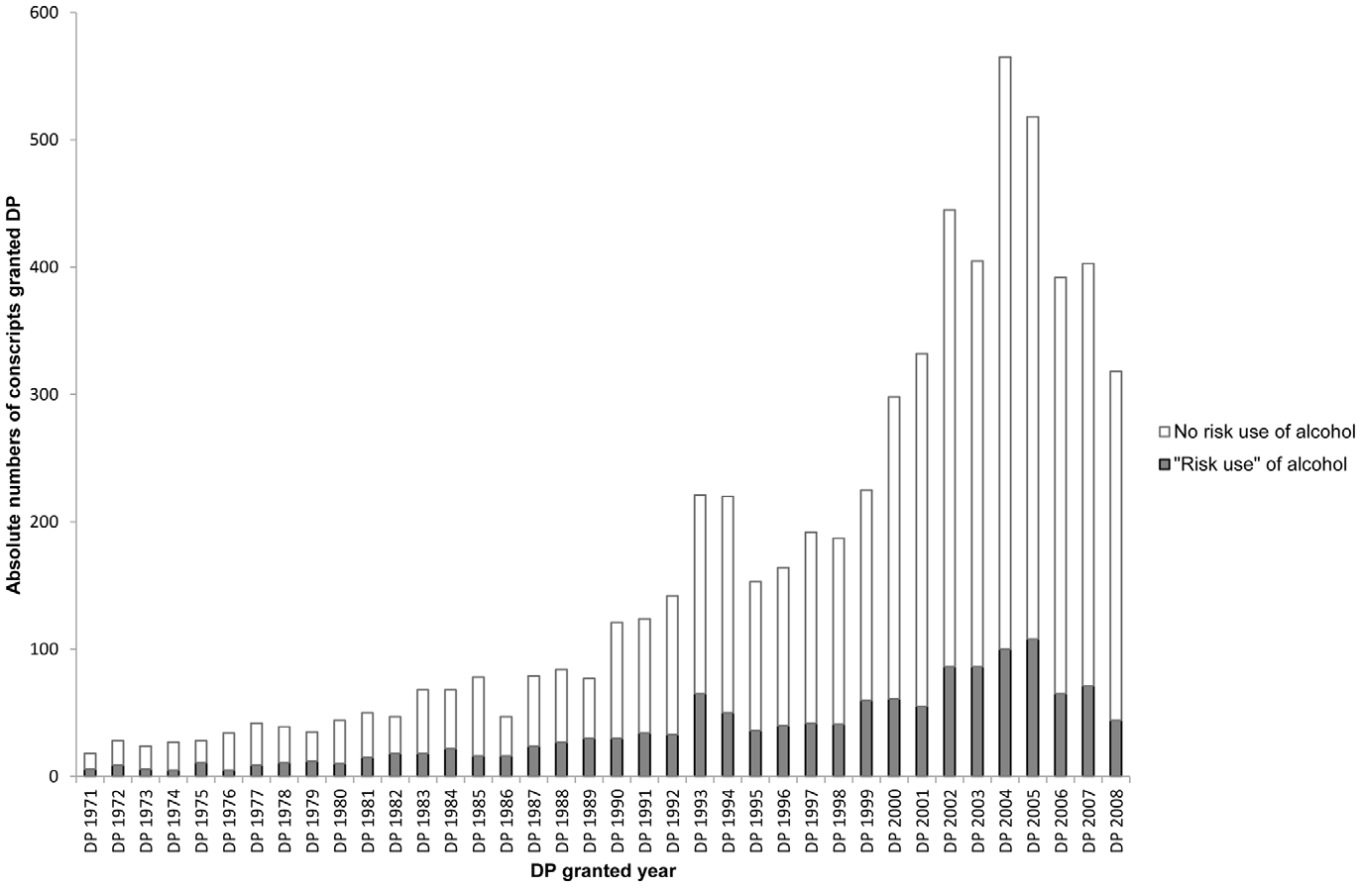
Conscripts granted disability pension in 1971–2008 and “risk users”^a^ of alcohol. Number of Swedish male conscripts granted disability pension (DP) in 1971–2008 and among them number of “risk users”^a^ of alcohol defined at conscription in 1969–1970. ^a^Subjects were classified as having “risk use” if at least one of the following condition was fulfilled: having a history of being apprehended for drunkenness, needing an eye-opener to overcome hangover, having been drunk often/quite often, reporting alcohol consumption measured as more than 250 g 100% alcohol/week.

Figure 1 shows that the number of DP granted over the years increased with age. The decline seen after the year 2004 is in line with the national trend in granting DP in Sweden in the mid-2000s [25]. Altogether 6342 conscripts were granted DP of whom 1038 persons received DP in 1970–1990 and 5304 conscripts after 1991. Figure 1 also shows that the proportion of “risk users” among disability pensioners slightly declined over time.

The measurement levels of each covariate and its distribution among the conscripts with different levels of exposures were presented in the previous articles based on the same cohort [22], [51], where a J-shaped association between the levels of consumption and variables such as social and behavior characteristics, physical and mental health, psychological conditions and sociability was observed. In the other words, prevalence of unfavorable status of several risk factors among abstainers was higher than among light consumers. Non-responders, i.e. those providing no answers on exposures of interest, differed from responders with respect to most of the covariates and DP was more prevalent in non-responders for both exposures with p<0.001 in both cases (data not shown). In univariate analysis each explanatory variable was positively associated with outcomes of interest (data not shown).

For different levels of consumption, crude analysis showed a strong association with DP (Table 3). After adjusting for each set of variables, one at the time, the increased HR persisted for all consumption levels with few exceptions. In Model 1 there was a positive association for DP with abstainers, while in Model 2 an increased risk of DP was seen for all consumption levels with the strongest associations for high consumers, which was even more obvious in Model 3. The strongest association was seen for “early DP”.

**Table 3:** Crude and adjusted HR (95% CI) for disability pension (DP) in total and for “early DP” granted in 1971–1990 and “late DP” granted in 1991–2008 in association with different levels of alcohol consumption measured in grams of 100% alcohol per week reported at conscription in 1969–1970 among 38 671 Swedish male conscripts with information available on all variables in the table.

	DP in total	“Early DP”	“Late DP”
Approximate age (years)	20–59	20–41	40–59
Variables controlled for	HR (95% CI)	HR (95% CI)	HR (95% CI)
**Crude**			
- 0 (g 100% alcohol/week) abstainers	1.30 (1.16–1.46)	2.31 (1.81–2.95)	1.14 (1.00–1.31)
- 1–100 light consumers (ref group)	1.0	1.0	1.0
- 101–250 moderate consumers	1.32 (1.23–1.41)	1.60 (1.34–1.89)	1.27 (1.18–1.37)
- >250 high consumers	2.25 (2.00–2.53)	3.37 (2.61–4.36)	2.06 (1.81–2.35)
**Adjusted for the sets of variables below (one set at the time)**			
Family background-related exposures^a^			
- 0	1.31 (1.17–1.48)	2.33 (1.82–2.98)	1.15 (1.01–1.32)
- 1–100	1.0	1.0	1.0
- 101–250	1.28 (1.19–1.37)	1.53 (1.28–1.82)	1.24 (1.15–1.34)
- >250	2.11 (1.87–2.37)	3.02 (2.33–3.92)	1.95 (1.70–2.22)
School-related exposures^b^			
- 0	1.34 (1.20–1.51)	2.46 (1.93–3.14)	1.17 (1.03–1.34)
- 1–100	1.0	1.0	1.0
- 101–250	1.26 (1.18–1.35)	1.45 (1.22–1.73)	1.23 (1.14–1.33)
- >250	2.09 (1.86–2.35)	2.89 (2.23–3.76)	1.94 (1.70–2.22)
Social and behavior exposures^c^			
- 0	1.38 (1.23–1.55)	2.51 (1.96–3.20)	1.21 (1.06–1.38)
- 1–100	1.0	1.0	1.0
- 101–250	1.12 (1.05–1.20)	1.23 (1.03–1.47)	1.10 (1.02–1.19)
- >250	1.51 (1.34–1.71)	1.76 (1.34–2.31)	1.46 (1.27–1.67)
Physical and mental health at conscription^d^			
- 0	1.23 (1.09–1.38)	2.03 (1.59–2.59)	1.09 (0.96–1.25)
- 1–100	1.0	1.0	1.0
- 101–250	1.20 (1.12–1.29)	1.32 (1.11–1.57)	1.18 (1.10–1.28)
- >250	1.56 (1.39–1.76)	1.70 (1.31–2.22)	1.53 (1.34–1.75)
Psychological status and sociability at conscription^e^			
- 0	1.17 (1.04–1.32)	1.83 (1.43–2.34)	1.05 (0.92–1.20)
- 1–100	1.0	1.0	1.0
- 101–250	1.17 (1.09–1.25)	1.27 (1.07–1.51)	1.15 (1.07–1.24)
- >250	1.52 (1.35–1.71)	1.69 (1.30–2.19)	1.48 (1.29–1.69)
Substance use at conscription^f^			
- 0	1.61 (1.43–1.82)	2.91 (2.25–3.76)	1.41 (1.23–1.62)
- 1–100	1.0	1.0	1.0
- 101–250	1.08 (1.01–1.16)	1.24 (1.04–1.49)	1.06 (0.98–1.14)
- >250	1.62 (1.43–1.84)	2.20 (1.66–2.91)	1.52 (1.32–1.74)
**Model 1** g			
- 0	1.34 (1.19–1.52)	1.95 (1.51–2.52)	1.22 (1.07–1.40)
- 1–100	1.0	1.0	1.0
- 101–250	0.99 (0.92–1.07)	1.06 (0.88–1.28)	0.98 (0.91–1.06)
- >250	1.05 (0.92–1.19)	1.06 (0.79–1.41)	1.05 (0.91–1.21)
**Model 2** h			
- 0	1.20 (1.07–1.35)	1.90 (1.48–2.43)	1.08 (0.95–1.23)
- 1–100	1.0	1.0	1.0
- 101–250	1.13 (1.05–1.21)	1.20 (1.00–1.43)	1.12 (1.04–1.21)
- >250	1.44 (1.27–1.62)	1.51 (1.16–1.98)	1.41 (1.23–1.62)
**Model 3** i			
- 0	1.35 (1.20–1.51)	2.45 (1.92–3.13)	1.18 (1.03–1.34)
- 1–100	1.0	1.0	1.0
- 101–250	1.23 (1.15–1.32)	1.41 (1.18–1.68)	1.20 (1.11–1.30)
- >250	1.98 (1.75–2.23)	2.64 (2.02–3.44)	1.85 (1.61–2.11)

a Corresponds to father’s socioeconomic position, father’s drinking habits, and parental divorce.

b Corresponds to truancy and remedial class.

c Corresponds to contact with police and childcare authorities, ever run away from home, and being unemployed for more than 3 months after finishing school.

d Corresponds to self-assessed health, medication to nervous problems, and any psychiatric diagnosis reported/detected at conscription.

e Corresponds to emotional control, social maturity, and cognitive ability assessed at conscription.

f Corresponds to smoking, sniffing of solvents, and drug use reported at conscription.

g Adjusted for all covariates in the table.

h Adjusted for family background, school-related exposures and exposures related to psychological status and sociability.

i Adjusted for family background and school-related exposures.

There was a strong association between “risk use” and DP regardless of adjustment and the highest risk was again seen for “early DP” (Table 4).

**Table 4:** Crude and adjusted HR (95% CI) for disability pension (DP) in total and for “early DP” granted in 1971–1990 and “late DP” granted in 1991–2008 in association with different characteristics of “risk use” of alcohol behaviors established at conscription in 1969–1970 among 38 899 Swedish male conscripts with information available on all variables in the table.

	DP in total	“Early DP”	“Late DP”
Approximate age	20–59	20–41	40–59
Variables controlled for	HR (95% CI)	HR (95% CI)	HR 95% CI
**Crude**			
- No “risk use” of alcohol (ref group)	1.0	1.0	1.0
- “Risk use” of alcohol	2.02 (1.89–2.17)	2.89 (2.47–3.38)	1.87 (1.74–2.02)
**Adjusted for the sets of variables below (one set at the time)**			
Family background-related exposures^a^	1.88 (1.76–2.02)	2.64 (2.25–3.10)	1.75 (1.62–1.89)
School-related exposures^b^	1.94 (1.81–2.07)	2.66 (2.27–3.12)	1.81 (1.67–1.95)
Social and behavior exposures^c^	1.46 (1.35–1.58)	1.81 (1.51–2.16)	1.39 (1.28–1.52)
Physical and mental health at conscription^d^	1.66 (1.55–1.78)	1.93 (1.63–2.27)	1.60 (1.48–1.73)
Psychological status and sociability at conscription^e^	1.51 (1.40–1.62)	1.71 (1.45–2.01)	1.46 (1.35–1.58)
Substance use at conscription^f^	1.68 (1.56–1.80)	2.37 (2.00–2.81)	1.56 (1.44–1.69)
**Model 1** g	1.17 (1.08–1.27)	1.32 (1.09–1.59)	1.14 (1.05–1.25)
**Model 2** h	1.44 (1.34–1.55)	1.61 (1.36–1.90)	1.40 (1.29–1.51)
**Model 3** i	1.81 (1.69–1.94)	2.46 (2.09–2.89)	1.70 (1.57–1.83)

a Corresponds to father’s socioeconomic position, father’s drinking habits, and parental divorce.

b Corresponds to truancy and remedial class.

c Corresponds to contact with police and childcare authorities, ever run away from home, and being unemployed for more than 3 months after finishing school.

d Corresponds to self-assessed health, medication to nervous problems, and any psychiatric diagnosis reported/detected at conscription.

e Corresponds to emotional control, social maturity, and cognitive ability (IQ) assessed at conscription.

f Corresponds to smoking, sniffing of solvents, and drug use reported at conscription.

g Adjusted for all covariates in the table.

h Adjusted for family background, school-related exposures and exposures related to psychological status and sociability.

i Adjusted for family background and school-related exposures.

Further subdivision within the group of abstainers revealed that 80% (2 218 out of 2 781) of those reporting never drinking any alcohol also reported abstention from use of other substances. However, a J-shaped curve was found in the distribution of medical, social and behavioral risk factors for “pure abstainers” as well as for “other abstainers” compared to other levels of consumption similar to that seen for the entire cohort. For example, 31% of “pure abstainers” and 41% of “other abstainers” were found to have the lowest level of emotional control, while only 26% of light alcohol consumers were found in this stratum. For 31% and 34% of “pure” and “other” abstainers, respectively, the level of social maturity was defined as “low and very low” compared to 19% of light consumers.

The results of Table 3 remained virtually unchanged when abstainers were analyzed as two sub-groups in crude analysis. For “early DP” adjusted HRs and 95% CIs also appeared to be similar to those seen in Table 3. For “late DP”, however, the adjusted results were less consistent. Thus, for “other abstainers” the increased risk persisted in all models, while for “pure abstainers” only Model 3 revealed a significant association (data not shown). In addition, when abstainers were excluded from the analysis of DP and “risk use” of alcohol, the associations previously seen in Table 4, remained significant and became stronger (data not shown).

Stratification by each covariate revealed only slight effect modification from some of the variables. Inclusion of interaction terms did not alter the results (data not shown).

The robustness of the results was tested in sensitivity analysis with all 49 321 conscripts included, showing no difference in crude associations with the results from Tables 3 and 4. Adjustment for one set of variables at the time also did not alter the results seen in the main analysis.

## Discussion

We found that alcohol use in adolescence is associated with an increased risk of DP, in particular “early DP”. “Risk users” of alcohol have a statistically significant increased risk regardless of adjustment. Controlling for family background and school-related exposures slightly attenuated the increased risk, while more pronounced reduction was observed when all covariates were controlled for; though the risk persisted in all models. Moderate and high consumers also had a statistically significant increased risk of DP, which gradually reduced when expanding the number of controlled covariates and disappeared when the most conservative model for adjustment had been used. Our findings are consistent with previous results where high alcohol consumption [4], [6], [52]–[56], hazardous drinking [4], [5], [52], and alcohol dependence and abuse [3] were positively associated with future DP and/or sickness absence. While our results confirm the findings from the 22-year follow-up of the same cohort [1], [2], it is of interest that the associations remain above the age of 40 and after thorough adjustment for potential confounders. The fact that a few studies did not find an increased risk for alcohol exposure might be due to differences in age and in rules for granting DP as well as to differences in studied diagnostic categories [7]–[9].

### DP Among Abstainers

The J-shaped distribution of alcohol consumption related to social and emotional maturity and physical and mental health puts our findings in line with previous results from this and other cohorts and it is similar to the J-shaped curve for the relation between alcohol consumption and mortality, morbidity and DP [4]–[6], [21], [22]. In fact, our findings are very similar to those reported by Månsson et al. [6], where the adjusted RRs for abstainers was the same as for those with the highest consumption level measured by serum gamma glutamyl transferase (RR of 1.8). In the studies by Månsson et al. [6] and Skogen et al [5], a plausible explanation put forward is the presence of “sick quitters” among abstainers, for which they found some support, but both authors conclude that more information is needed on characteristics of abstainers to understand the increased risk of DP in this group. In our population of young men, the likelihood of persons having stopped using alcohol due to illness is less probable. Important characteristics that may explain part of the phenomenon can be found in an earlier study on this cohort focusing on psychosocial and behavior characteristic of the conscripts [22]. Abstainers scored higher than moderate consumers on several indicators of poor sociability: insecure in company of others, unpopular in school, never intimate conversations with friends, etc. [22]. Abstainers also had low degree of emotional control and higher prevalence of psychiatric diagnoses [22]. All these are factors that have been shown to be related to increased mortality and morbidity in this cohort [1], [2], [28], [34]. Already Vaillant GE [57] found a U-shaped relationship between alcohol use and mental health, with abstainers and alcohol abusers showing the highest scores. Tucker et al. [58] showed somewhat different results regarding drug use among adolescents in the sense that abstainers often fared better than experimenters and frequent users. Our cohort of young men grew up in a period with a liberal attitude to alcohol, but less so to drug use. Use of marijuana and other drugs among adolescents has been less common in Sweden, while use of alcohol was common and more part of social norm, especially among young people in the end of the 60 s [22].

### The Role of Pattern of Drinking

“Risk use” of alcohol was more strongly associated with DP regardless of adjustment than high consumption. A similar relation was seen in the Norwegian HUNT study [5].

The complex association between pattern of drinking and various health outcomes was highlighted by Room et al. [59], and WHO [17]. It was shown that drinking pattern may be an even more appropriate measure of alcohol use on the individual level and in the society compared to one-dimensional measure of the level of consumption [17], [59].

### Methodological Issues

A major methodological challenge deals with the complicated nature of associations between reported social, mental and behavioral conditions and alcohol use. We could not test for mediation since data on exposures and covariates were collected at one point of time and the temporal order for some variables was not clear. Thus, we could not definitely conclude that the risk reduction is due to mediation. A pragmatic approach was, therefore, to study the association in three models, in which we can assume that our most conservative model is more likely to result in overadjustment, since some of the variables may act as mediators [37]. The reduction was stronger for levels of consumption, while “risk use” seemed to be less related to other covariates. Examination of the possible pathways of alcohol consumption in relation to social and behavioral risk factors in adolescence may help us to understand the phenomenon. It is possible that the causal chain between levels of consumption and DP differs from the one seen for the more complicated “risk use” measure of alcohol exposure and confounding/mediating effect of variables may also differ in strength.

### Strengths and Limitations

One of the major strengths of this study is the size that ensures considerable statistical power. Due to the high response rate, the population-based nature of the cohort and availability of data from numerous registers, the information on DP, drinking habits and levels of consumption as well as on various medical and social conditions was available for almost each conscript. The completeness of the follow-up data in the Swedish social insurance databases minimizes any bias due to selective non-response by problem drinkers or poor registration of outcome.

One limitation is that the study only concerns men. Another important limitation is lack of data on alcohol consumption later in life. It has, however, been previously shown on the same material that there is a strong association between levels of alcohol reported at conscription and later alcohol-related hospitalization [19] and alcohol-related death [33] that, at least, partly must be due to continuation of early life drinking habits. The third limitation is the lack of data on diagnoses for DP. It would be of interest to find out whether the relation between DP and alcohol use varies with DP diagnostic categories.

In addition, we acknowledge that self-reported data on alcohol use may be a subject for underreporting, even though in studies on youngsters, overreporting can also be present [51]. Several studies on conscripts in Sweden [1], [51], [60], [61] have addressed this issue and proven reliability of this type of data. Also information bias of exposure, if any, is likely to lead to non-differential misclassification as it does not depend on the status of outcome and, thus, might only result in underestimation of association of interest [37]. Similar to this study, others found a higher prevalence of DP [62], higher incidence and prevalence of alcohol dependency and overconsumption [62], [63] and higher mortality rate [62] among non-participants compared to participants. However, this does not imply that the association between the studied exposures and DP is different for the non-participants.

We conclude that the early life exposure to alcohol is associated with increased risk of future DP. The association is particularly strong for “early DP” but remains significant among those granted DP up to the age of 59 emphasizing the public health and socio-economic burden of the outcome. Pattern of drinking was a more pronounced risk factor for DP and, therefore, drinking behavior in adolescent should be considered an important marker for future work incapacity.

Our findings add knowledge on the health consequences of early alcohol use. In addition to early mortality and morbidity, early alcohol use is an indicator of future work problems and exclusion from the labor market.
